# Comparison of clinical effects and patient satisfaction between ultra-pulsed CO₂ laser treatment and surgical excision in patients with facial basal cell carcinoma

**DOI:** 10.3389/fsurg.2025.1628257

**Published:** 2025-08-14

**Authors:** An-Chen Chen, Chen Zheng, Qi-Chao Jian, Peng Zhang

**Affiliations:** Department of Dermatology, Huangshi Central Hospital, Affiliated Hospital of Hubei Polytechnic University, Huangshi, Hubei, China

**Keywords:** ultra-pulsed CO₂ laser treatment, basal cell carcinoma, surgical excision, clinical outcomes, patient satisfaction

## Abstract

**Background:**

Basal cell carcinoma (BCC) commonly affects facial skin, with surgical excision being the usual treatment. However, surgery often leads to complications and slow healing, impacting quality of life. Recently, ultra-pulsed CO₂ laser has emerged as a minimally invasive option with good cosmetic results, but its effectiveness and patient satisfaction compared to surgery are still uncertain.

**Objective:**

This study aims to compare the clinical outcomes and patient satisfaction between ultra-pulsed CO₂ laser treatment and surgical excision for patients with facial BCC.

**Methods:**

A retrospective analysis was conducted on 100 patients with facial BCC treated at our dermatology department from January 2021 to January 2024.Among them, 50 patients received ultra-pulsed CO₂ laser treatment, while 50 underwent traditional surgical excision. We compared the tumor excision rates, incidence of postoperative complications, healing times, and patient satisfaction (assessed through a questionnaire) between the two groups.

**Results:**

The clinical effective rate in the ultra-pulsed CO₂ laser group was 94.0%, compared to 90.0% in the surgical excision group, with no statistically significant difference between the two groups (*p* > 0.05).Postoperative complications in the CO₂ laser group were primarily mild burns and inflammatory erythema, all of which resolved spontaneously within 1–2 days without treatment, with no serious adverse reactions reported. Recurrence rates were 4.00% (laser) vs. 16.00% (surgery), the difference between the two groups was statistically significant (*p* < 0.05). Regarding pain scores, there was no significant difference in preoperative pain scores between the groups; however, the CO₂ laser group reported significantly lower pain scores at 1, 3 days and 7days postoperatively (*P* < 0.001). Furthermore, patient satisfaction was significantly higher in the CO₂ laser group compared to the surgical group (96.0% vs. 76.0%, *P* < 0.001).

**Conclusion:**

In summary, both ultra-pulsed CO₂ laser treatment and surgical excision exhibit similar clinical efficacy in the management of facial basal cell carcinoma. Nonetheless, ultra-pulsed CO₂ laser treatment offers notable benefits regarding postoperative complication rates, pain scores, and patient satisfaction. Consequently, ultra-pulsed CO₂ laser treatment may be regarded as an effective and patient-friendly alternative for the treatment of facial basal cell carcinoma.

## Introduction

Basal cell carcinoma (BCC) is one of the most common skin malignancies, particularly prevalent in facial regions (1). BCC arises from the atypical proliferation of cells within the basal layer of the epidermis. Owing to its indolent growth and minimal metastatic potential, it is frequently categorized as a relatively benign neoplasm (2). However, with the aging global population and increased ultraviolet exposure, the incidence of BCC has been rising annually, significantly impacting patients' quality of life (3). This is particularly applicable to lesions situated in prominent areas, such as the face, where they not only impact aesthetic appearance but may also contribute to psychological distress and social anxiety (4).

Surgical excision has traditionally been considered the primary approach for treating BCC (5). By directly removing the tumor along with surrounding tissue, surgical excision effectively eliminates tumor cells and reduces the risk of recurrence (6). However, this procedure is frequently linked to a greater risk of postoperative issues, such as infections, excessive bleeding, pain, and the formation of scars (7). These complications can extend the recovery period and adversely affect patients' mental health and social interactions (8). Moreover, the extended healing time post-surgery further increases the burden on patients.

Recent advancements in laser technology have sparked significant interest in ultra-pulsed CO₂ laser treatment, primarily due to its minimally invasive properties and excellent cosmetic results (9). This technique employs a high-energy laser beam to precisely excise tumor tissue while minimizing damage to surrounding healthy tissue, thereby reducing the risk of postoperative complications and accelerating the healing process (10). When compared to traditional surgical excision, ultra-pulsed CO₂ laser treatment presents several advantages, including reduced trauma, quicker recovery times, and enhanced aesthetic outcomes—factors that are leading to its growing acceptance among patients and healthcare providers alike (11).

While various studies have investigated the use of laser therapy for treating skin tumors, there is still a notable gap in comparative research focusing on clinical outcomes and patient satisfaction between ultra-pulsed CO₂ laser treatment and traditional surgical excision for BCC. This study seeks to systematically assess the clinical effectiveness and patient satisfaction associated with ultra-pulsed CO₂ laser treatment compared to surgical excision in individuals with facial BCC. The results aim to deliver more precise and trustworthy evidence for clinical practice, assisting physicians in creating more informed and effective treatment strategies for their patients.

## Methods

### General information

This study conducted a retrospective analysis of 100 patients with facial BCC who received treatment in the dermatology department of our hospital from March 2021 to October 2024. All patients were diagnosed through pathology and had no significant comorbidities. Participants were categorized into two groups based on their treatment approach: the ultra-pulsed CO₂ laser treatment group, which included 50 patients, and the surgical excision group, also comprising 50 patients. Group allocation was determined by clinical decision-making considering tumor characteristics and patient preference, ensuring no significant differences in baseline data.

Specifically, several factors influenced the group assignment:

• Tumor site: For tumors located in cosmetically sensitive areas (such as the nasal tip, eyelid margin, and lip), where preserving facial appearance is of high priority, patients were more likely to be assigned to the ultra-pulsed CO₂ laser treatment group after full communication with physicians. In contrast, tumors in non-cosmetically critical areas (such as the forehead, cheek with abundant soft tissue) were more inclined to be treated with surgical excision.
• Patient preference: Some patients expressed strong willingness to avoid surgical scars or preferred a less invasive treatment, thus choosing ultra-pulsed CO₂ laser treatment; others preferred a more radical treatment method and opted for surgical excision.
• Tumor characteristics: Although all tumors had a diameter of 2.0 cm or less, for those with a relatively shallow invasion depth (assessed by preoperative ultrasound), ultra-pulsed CO₂ laser treatment was more often recommended, while surgical excision was preferred for tumors with a slightly deeper invasion.

### Inclusion and exclusion criteria

Inclusion criteria were as follows:

1. Confirmation of basal cell carcinoma through skin biopsy and histological examination (12);
2. Patients had not received any other treatments such as radiotherapy or chemotherapy;
3. Tumor diameter was 2.0 cm or less;
4. Availability of complete clinical data.

Exclusion criteria included:

1. History of photosensitivity;
2. Presence of severe systemic diseases;
3. Currently undergoing radiotherapy or chemotherapy;
4. Pregnant or breastfeeding women.

### Ethical approval

This study was reviewed and approved by the Ethics Committee of Huangshi Central Hospital. A formal waiver of informed consent for study participation was granted by the committee due to the retrospective nature of the research, which involved analysis of de-identified clinical data routinely collected during standard care. All patient information was processed with strict confidentiality measures to protect privacy, in accordance with the Declaration of Helsinki and local ethical guidelines. As no identifiable personal data or images were included in the manuscript, additional consent for publication was not required.

### Treatment methods

Ultra-pulsed CO₂ Laser Treatment (13): Patients received ultra-pulsed CO₂ laser treatment under local anesthesia. The specific steps are as follows: the lesion area was routinely disinfected, and local infiltration anesthesia (2% lidocaine) was administered. A CO₂ laser treatment machine was used to ablate the diseased tissue. The laser was set with a wavelength of 10.6 μm, pulse duration of 100–300 μs, and fluence of 50–100 J/cm^2^. Initially, the tumor was vaporized from the epidermis gradually deeper into the lesion. At a distance of 0.5 cm from the lesion edge, coagulation vaporization was performed around the lesion until reaching the dermal papilla layer. Upon observing the dermal papilla granules, vaporization was continued inward along the dermis, surrounding the tumor until complete removal of the lesion. If bleeding occurred during vaporization, coagulation hemostasis was performed first, followed by vaporization. The 635 nm semiconductor laser treatment device was manufactured by Wuhan Lingyun Optoelectronic Technology Co., Ltd., model FD-400-B; the CO₂ laser treatment machine was produced by Xiaogan Sunshine Shenqi Medical Technology Co., Ltd., model YG-40A.

Surgical Excision: Patients in the surgical group underwent traditional surgical excision under local anesthesia. The specific steps are as follows: local anesthesia was administered using 2% lidocaine, and a diamond-shaped excision was performed at a distance of 5 mm from the tumor edge, removing the basal cell carcinoma and an additional 5 mm of surrounding skin. The subcutaneous tissue was incised sequentially until reaching the fat layer. After excising the tumor and surrounding tissue, the wound was irrigated with saline, and the area was sutured after confirming that the lesion had been completely excised.

### Observational indicators

Basic patient information was collected, including age, sex, tumor size, and location.

1. Clinical Efficacy Rate (14): All patients were followed up one year after treatment to assess the morphology, color, and area of the lesions and to evaluate efficacy. Complete response was defined as the disappearance of the lesion, with only pigmentation or depigmentation remaining, and no original pathological changes on histological examination; partial response was defined as a reduction of 50% or more in the lesion size; no response was defined as a reduction of less than 50% or no change. The total efficacy rate was the sum of complete and partial responses.
2. Complication Incidence: The incidence of complications occurring within 30 days postoperatively was recorded for both groups, including infection, bleeding, and scar formation.
3. Postoperative Recurrence Rate: All patients were followed up for one year after treatment. Recurrence was defined as the appearance of new lesions at the site of the previously disappeared lesion.
4. Postoperative Pain Assessment (15): Pain was assessed using the Visual Analog Scale (VAS), where 0 indicated no pain, 1–3 indicated mild pain, 4–6 indicated moderate pain, 7–9 indicated severe pain, and 10 indicated unbearable pain. Pain assessments were conducted preoperatively and on postoperative days 1, 3, and 7.
5. Patient Satisfaction: Assessed at 3-month follow-up, Patient satisfaction was evaluated at the 3-month postoperative follow-up using a study-specific questionnaire. The satisfaction questionnaire was developed based on clinical aesthetic concerns of facial BCC patients, including 3 core items: scar visibility, impact on facial appearance, and overall acceptability. It was categorized as follows: satisfied if only a linear scar remained and the patient felt it did not affect aesthetics; generally satisfied if the scar was more noticeable and mildly affected aesthetics; dissatisfied if a large scar remained and significantly affected aesthetics.

### Statistical analysis

Statistical analyses were performed using IBM SPSS Statistics version 26.0 (IBM Corp., Armonk, NY, USA). Continuous data were expressed as mean ± standard deviation (SD) and analyzed using independent samples *t*-tests or paired *t*-tests, as appropriate. Categorical variables were presented as counts (percentages). For comparisons of proportions between two independent groups, Pearson's chi-square test was used when all expected frequencies were ≥5; otherwise, Fisher's exact test was applied. A two-sided *p* < 0.05 was considered statistically significant.

## Results

### Comparison of baseline data between groups

In the CO₂ laser group of 50 patients, 66.0% (33 cases) were male and 34.0% (17 cases) were female, with an average age of 67.5 ± 13.2 years. In the surgical group, 70.0% (35 cases) were male and 30.0% (15 cases) were female, with an average age of 67.7 ± 11.5 years. The maximum diameter of the lesions in the CO₂ laser group was 1.68 ± 0.74 cm, while in the surgical group it was 1.81 ± 0.93 cm. In the CO₂ laser group, 76.0% (38 cases) were patients with sun exposure, compared to 74.0% (37 cases) in the surgical group. Statistical analysis showed no significant differences in baseline data between the two groups (*P* > 0.05), indicating comparability, as shown in Table 1.

**Table 1:** Description of the study population and main clinical features of the treated lesions.

Patients	CO₂ laser (*n* = 50)	Surgery (*n* = 50)	*P*
Gender			
Male	33 (66.0)	35 (70.0)	0.136
Female	17 (34.0)	15 (30.0)	
Age (mean ± SD), years	67.5 ± 13.2	67.7 ± 11.5	0.842
Phototype			
I	7 (14.0)	8 (16.0)	0.381
II	6 (12.0)	7 (14.0)	
III	24 (48.0)	25 (50.0)	
IV	13 (26.0)	10 (20.0)	
Maximal diameter (mean ± SD), cm	1.68 ± 0.74	1.81 ± 0.93	0.263
Regarding sun exposure			
Sun exposed patients	38 (76.0)	37 (74.0)	0.349
Not sun exposed patients	12 (24.0)	13 (26.0)	
Sites of the lesions			
Nose	18 (36.0)	19 (38.0)	0.572
Scalp	12 (24.0)	13 (26.0)	
Ear	10 (20.0)	8 (16.0)	
Forehead	8 (16.0)	6 (12.0)	
Medial canthus	2 (4.0)	4 (8.0%)	

SD, standard deviation.

### Overall clinical efficacy rate

One year after treatment, the overall clinical efficacy was compared between the two groups. In the CO₂ laser group, out of 50 patients, 45 achieved complete response and 2 achieved partial response, resulting in an overall efficacy rate of 94.0% (47/50). In the surgical group, out of 50 patients, 40 achieved complete response and 5 achieved partial response, leading to an overall efficacy rate of 90.0% (45/50). The difference in overall efficacy rates between the two groups was not statistically significant (*P* > 0.05), as detailed in Table 2.

**Table 2:** Comparison of efficacy between two groups, *n* (%).

Group	CO₂ laser (*n* = 50)	Surgery (*n* = 50)	*P*
Complete remission	45	40	
Partial response	2	5	
No response	3	5	
Total effect rate (%)	94.0%	90.0%	>0.05

### Incidence of complications

After treatment, all patients in the CO₂ laser group experienced mild burns and inflammatory erythema, which resolved spontaneously within 1–2 days without serious adverse reactions. In the surgical group, 2 cases developed significant scar formation that affected the patients' appearance.

### Postoperative recurrence rate

In the CO₂ laser group, there was 2 cases of recurrence, resulting in a recurrence rate of 4.00%. In the surgical group, there were 8 cases of recurrence, leading to a recurrence rate of 16.00%. The difference in overall recurrence rates between the CO₂ laser group and surgical group was statistically significant (*P* < 0.05), as illustrated in Table 3.

**Table 3:** Postoperative recurrence rates by treatment group.

Group	n	Recurrence cases, *n* (%)	*P*
CO₂ laser	50	2 (4.00%)	<0.05
Surgery	50	8 (16.00%)	

### Comparison of VAS scores between groups

The preoperative pain score in the CO₂ laser group was 4.58 ± 1.23, compared to 4.52 ± 1.05 in the surgical group, with no statistically significant difference (*P* > 0.05). The postoperative pain scores on day 1 (1.31 ± 0.42) and day 3 (0.22 ± 0.14) were both lower than those in the control group (2.48 ± 0.37 and 1.69 ± 0.35, respectively), with statistically significant differences (*P* < 0.001), By day 7, pain scores in both groups were low (CO₂ laser: 0.08 ± 0.05; Surgical: 0.41 ± 0.18), but the difference remained statistically significant (*P* < 0.001) as shown in Table 4.

**Table 4:** Comparison of VAS scores between the two groups.

VAS scores	CO₂ laser (*n* = 50)	Surgery (*n* = 50)	*P*
Before surgery	4.58 ± 1.23	4.52 ± 1.05	>0.05
1d after surgery	1.31 ± 0.42	2.48 ± 0.37	*P* < 0.001
3d after surgery	0.22 ± 0.14	1.69 ± 0.35	*P* < 0.001
7d after surgery	0.08 ± 0.05	0.41 ± 0.18	*P* < 0.001

VAS, visual analog scale.

### Comparison of surgical satisfaction between groups

**Figure 1:**
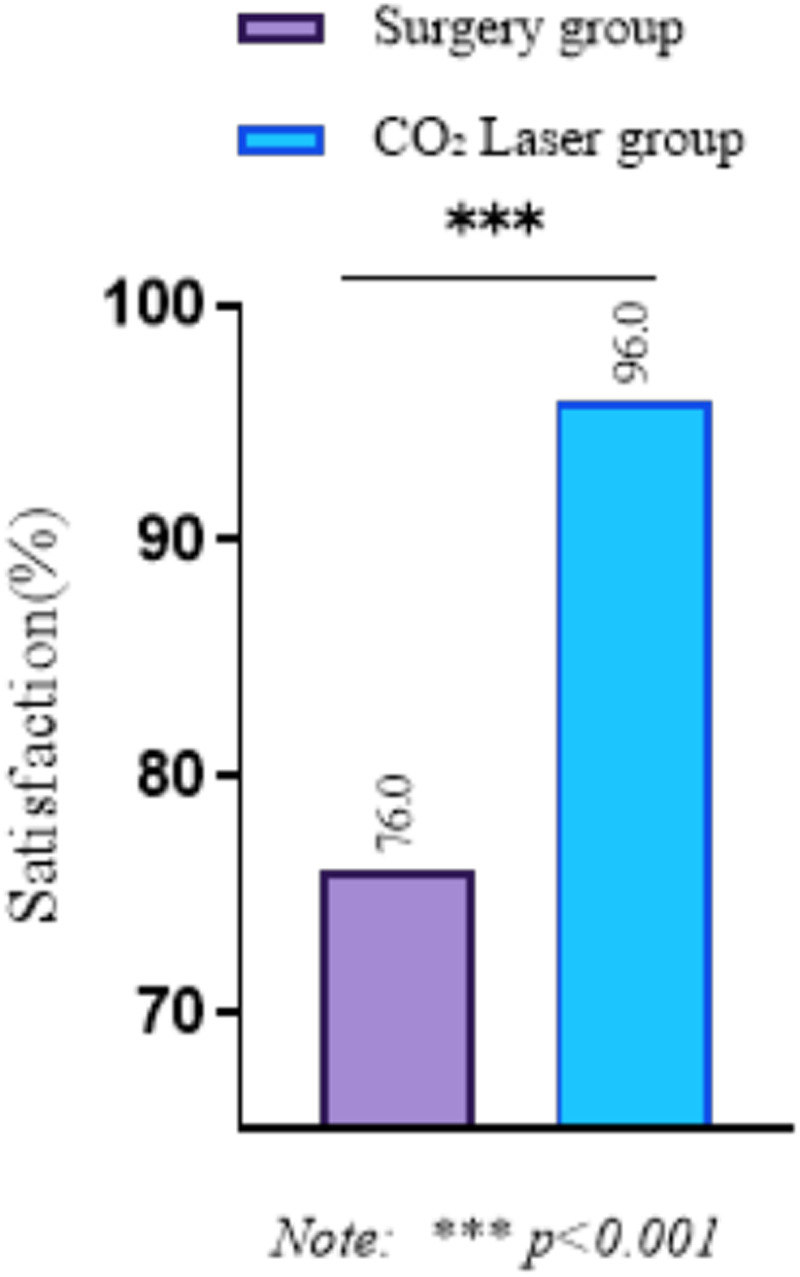
Comparison of satisfaction between the two groups of patients.

The surgical satisfaction rate in the CO₂ laser group was 96.0% (48/50), significantly higher than the surgical group's satisfaction rate of 76.0% (38/50). The difference was statistically significant (*P* < 0.001), as presented in Table 5 and Figure 1.

**Table 5:** Comparison of the satisfaction between the two groups.

Group	CO₂ Laser (*n* = 50)	Surgery (*n* = 50)	*P*
Satisfied	48	38	
Basically satisfied	1	2	
Dissatisfied	1	10	
Total satisfied rate (%)	96%	76%	<0.001

## Discussion

BCC is a common clinical skin tumor with an increasing incidence rate each year. The lesions are often superficial and grow slowly, with a low mortality rate (16). However, BCC exhibits characteristics of general malignant tumors, such as infiltration, invasion, and metastasis, which can lead to delays in treatment and serious adverse effects on patients' health (17). Currently, traditional surgery remains the primary method for treating BCC in clinical practice, but it often results in larger wounds and a higher likelihood of scarring.

This study compared the clinical effectiveness and patient satisfaction of ultra-pulsed CO₂ laser treatment vs. surgical excision for facial BCC. The results indicated no significant differences in baseline data between the two groups, ensuring the comparability and reliability of the study. In terms of overall clinical efficacy, both groups demonstrated high treatment effectiveness, with the CO₂ laser group achieving 94.0% and the surgical group achieving 90.0%. The difference in efficacy rates was not statistically significant (*P* > 0.05), suggesting that ultra-pulsed CO₂ laser treatment and surgical excision have comparable clinical effects in treating BCC (18).

The analysis revealed notable differences between the two groups in terms of complication rates, postoperative recurrence, pain scores, and overall satisfaction. In the CO₂ laser treatment group, postoperative complications were mainly minor, such as mild burns and inflammatory erythema, which typically resolved on their own within one to two days, without any serious side effects. In contrast, the surgical excision group reported two cases of significant scar formation that impacted aesthetics. Furthermore, the recurrence rate after surgery was considerably lower in the CO₂ laser group compared to the surgical group (4.00% vs. 16.00%, *P* < 0.05).

In terms of pain management, although preoperative pain scores were comparable between the two groups, the CO₂ laser treatment group demonstrated significantly lower pain scores on postoperative days 1, 3, and 7, highlighting the advantage of laser therapy in controlling postoperative discomfort. Additionally, patient satisfaction was markedly higher in the CO₂ laser group (96.0% vs. 76.0%, *P* < 0.001), reinforcing the benefits of this treatment option for BCC. These findings are consistent with previous studies that demonstrate the advantages of ultra-pulsed CO₂ laser treatment, including its minimally invasive nature, quicker recovery times, and improved cosmetic outcomes, alongside a reduced rate of complications (19–22). Additionally, other studies have noted the advantages of laser treatment in reducing postoperative pain and enhancing patient satisfaction (23–25). The outcomes confirm the study's conclusions, highlighting the effectiveness and benefits of ultra-pulsed CO₂ laser treatment for BCC.

## Limitations

While this study has yielded valuable insights, it does have several limitations. First, being a single-center, retrospective analysis with a relatively small sample size. Notably, the non-randomized assignment based on clinical factors, especially patient preference and cosmetic location, introduces substantial potential for selection bias, particularly concerning outcomes like patient satisfaction and potentially recurrence. Second, the clinical outcomes were only assessed one year post-treatment, lacking long-term follow-up on recurrence rates, which limits the ability to evaluate the lasting effects of both treatment options. Additionally, the study did not analyze or optimize specific parameters of the laser treatment, which could impact the accuracy and reproducibility of the results. The satisfaction instrument, though clinically grounded, requires formal validation in future studies. Although we extended pain assessment to postoperative day 7, more frequent measurements during the first 72 h might have revealed nuanced pain patterns. Future studies could incorporate daily assessments during acute recovery. Future research should focus on increasing the sample size, extending the follow-up period, and refining the parameters of laser treatment to provide a more thorough evaluation of the clinical outcomes associated with ultra-pulsed CO₂ laser therapy for BCC.

## Conclusion

In summary, both ultra-pulsed CO₂ laser treatment and surgical excision exhibit similar clinical efficacy in the management of facial BCC. Nevertheless, ultra-pulsed CO₂ laser treatment offers distinct benefits concerning the incidence of complications, postoperative recurrence rates, pain scores, and patient satisfaction. Consequently, ultra-pulsed CO₂ laser treatment may be regarded as an effective and patient-friendly alternative for the treatment of facial BCC. Future research should aim to further investigate and refine the specific parameters of laser treatment to improve therapeutic outcomes and enhance patient satisfaction.

## Data Availability

The raw data supporting the conclusions of this article will be made available by the authors, without undue reservation.
